# The Response of Cowpea (*Vigna unguiculata*) Plants to Three Abiotic Stresses Applied with Increasing Intensity: Hypoxia, Salinity, and Water Deficit

**DOI:** 10.3390/metabo12010038

**Published:** 2022-01-04

**Authors:** Jayamini Jayawardhane, Juran C. Goyali, Somaieh Zafari, Abir U. Igamberdiev

**Affiliations:** 1 Department of Biology, Memorial University of Newfoundland, St. John’s, NL A1B 3X9, Canada; Juran.Goyali@mi.mun.ca (J.C.G.); szafari@mun.ca (S.Z.); 2 Department of Botany, Faculty of Science, University of British Columbia, Vancouver, BC V6T 1Z4, Canada; 3 Centre for Aquaculture and Seafood Development, Fisheries and Marine Institute of Memorial University, St. John’s, NL A1C 5R3, Canada

**Keywords:** *Vigna unguiculata*, hypoxia, water deficit, salinity, antioxidants, adaptive response

## Abstract

Exposing plants to gradually increasing stress and to abiotic shock represents two different phenomena. The knowledge on plants’ responses following gradually increasing stress is limited, as many of the studies are focused on abiotic shock responses. We aimed to investigate how cowpea (*Vigna unguiculata* (L.) Walp.) plants respond to three common agricultural abiotic stresses: hypoxia (applied with the increasing time of exposure to nitrogen gas), salinity (gradually increasing NaCl concentration), and water deficit (gradual decrease in water supply). We hypothesized that the cowpea plants would increase in tolerance to these three abiotic stresses when their intensities rose in a stepwise manner. Following two weeks of treatments, leaf and whole-plant fresh weights declined, soluble sugar levels in leaves decreased, and lipid peroxidation of leaves and roots and the levels of leaf electrolyte leakage increased. Polyphenol oxidase activity in both roots and leaves exhibited a marked increase as compared to catalase and peroxidase. Leaf flavonoid content decreased considerably after hypoxia, while it increased under water deficit treatment. NO emission rates after 3 h in the hypoxically treated plants were similar to the controls, while the other two treatments resulted in lower values of NO production, and these levels further decreased with time. The degree of these changes was dependent on the type of treatment, and the observed effects were more substantial in leaves than in roots. In summary, the responses of cowpea plants to abiotic stress depend on the type and the degree of stress applied and the plant organs.

## 1. Introduction

Cowpea (*Vigna unguiculata* (L.) Walp.) is an important legume and a key crop in the context of global climate change and food security [1]. This species has become one of the eight grain legume crops being targeted for yield and agronomic improvement by the Consultative Group for International Agricultural Research (CGIAR) [2]. Most of the studies of cowpea are focused on active breeding to combat poverty in developing countries [3]. This species is used in sustainable farming for being relatively tolerant to drought, water deficit, high temperature, and high salinity, as well as having the ability to fix atmospheric nitrogen and to reduce soil erosion [1,2,4,5,6]. It represents an environmental friendly, stress-tolerant, and inexpensive source of protein in many countries, and is especially indispensable for vegetarian populations.

In this study, we analyzed three abiotic stresses common in agriculture namely, hypoxia/re-oxygenation, high salinity, and water deficit. Oxygen-deprived conditions such as flooding, or waterlogging create hypoxia or anoxia in plants [7]. Salinity has been recognized as the most adverse condition, above other environmental constraints, in terms of severe effects on plant growth and development, photosynthesis, and ion homeostasis [8] due to osmotic, ionic, and oxidative stresses [9]. Water stress is one of the limiting factors for grain production, plant growth, and metabolism [10]. Moreover, due to the increasing incidences of climate change around the world, studying how plants respond to stresses helps to screen their important morphological and physiological characteristics; this will help to identify important plant traits that could be improved to help them thrive better under changing environmental conditions. Although the study of biochemical and physiological mechanisms of cowpea following abiotic shock, applied on different individuals using the same experimental setup, has been pursued by several authors, a comprehensive investigation of the morphological, physiological, and biochemical parameters of this species following the gradual or stepwise increased stress is lacking.

Gradually increasing abiotic stress and sudden shock are two different phenomena showing different patterns of metabolic changes in plant cells. Gene expression profiles are smooth when the stress factor e.g., salt treatment is applied in a gradual manner, while the osmotic shock or sudden application of salt results in strong and rapid changes in the expression of several genes [11]. The majority of the experiments conducted on salinity involved the exposure of plants to sudden salt shock, where seedlings experienced severe osmotic abnormalities, leading to uncontrolled cell death [11,12]. This does not occur in reality under field conditions, because soil slowly becomes more saline as groundwater content is reduced gradually during summer or dry periods [12]. When plants are exposed to stepwise-increased stresses, they tend to acclimatize to the conditions gradually, which closely reflects the natural incidence of abiotic stress [12,13]. Essential differences in the metabolic regulation of plants have been recorded under mild and gradual salt stress as compared to salt shock [11,12]. In this study, we chose intensities of treatments that reflect and simulate naturally occurring changes in the environment to which plants have to adapt during their life cycle via acclimatization to stress and coping with injuries—conditions that are not achievable in the conventional stress–shock experiments [12].

Several factors or mechanisms operate independently or jointly to enable plants to cope with abiotic stresses, especially under high salinity, drought, or water deficit; their tolerance is manifested as a complex trait [14]. Fine adaptation mechanisms of cowpea plants to stresses include the modulation of transcription factors determining cell proliferation [15] and upregulation of the members of kinase families [16]. These regulatory mechanisms induce anatomical, physiological, and biochemical adjustments of plants that are considered to be an integrated response of different organs, especially roots and leaves [17]. Since plant growth and leaf photosynthetic capacity are the main processes affected by abiotic stress, the study of those characteristics under stress conditions helps to explain the acclimation of plants to the environment [18]. Abiotic stresses trigger the formation of toxic reactive oxygen species (ROS) and reactive nitrogen species (RNS) in plant cells, resulting in an oxidative burst that damages cell metabolism and growth [19,20]. To alleviate the oxidative damage, plants develop different defense mechanisms for instance, increasing the activities of antioxidant compounds and enzymes. Moreover, organic osmolytes such as free proline, sugars, and amino acids are accumulated in the cells for osmoregulation, especially under salt stress [8]. Therefore, we tested several morphological, physiological, and biochemical parameters to see how cowpea species respond to the gradual increase in the three abiotic stresses, with the aim of verifying the hypothesis that plants are able to increase their tolerance to abiotic stress when its intensity rises in a stepwise manner.

## 2. Results

### 2.1. Soil Parameters Following Treatments

Soil pH values increased considerably following hypoxia, while the conductivity and total dissolved solids decreased as compared to the controls. All soil parameters (pH, conductivity, total dissolved solids) increased drastically when the plants were treated with increasing salinity or decreasing water levels for two weeks (Table 1). Salt stress caused the largest increases in conductivity and total dissolved solids, which were 18 and 17 times higher in treated plants than in control plants, respectively.

### 2.2. Morphological and Physiological Parameters of Cowpea Plants Following Three Treatments

Root and shoot length, along with fresh and dry weight of leaves and of the whole plants, decreased significantly, while the plant water-use efficiency increased following gradually increased hypoxia and subsequent reaeration.

Following the salt stress treatment, shoot and root lengths, the number of compound leaves, the fresh weight of the leaves and of the whole plants, the dry weight of leaves, and the plant water-use efficiency decreased significantly, whereas stomatal density increased markedly (Table 2).

Water deficit decreased the number of compound leaves, the fresh weight of leaves and of the whole plants, the relative water content of leaves, and the plant water-use efficiency significantly, whereas root lengths, stomatal density, and stomatal index increased (Table 2). Other morphological and physiological parameters did not change significantly as compared to the controls.

### 2.3. Photosynthetic Pigments, Total Soluble Sugars, and Protein Content Following the Three Treatments

**Figure 1:**
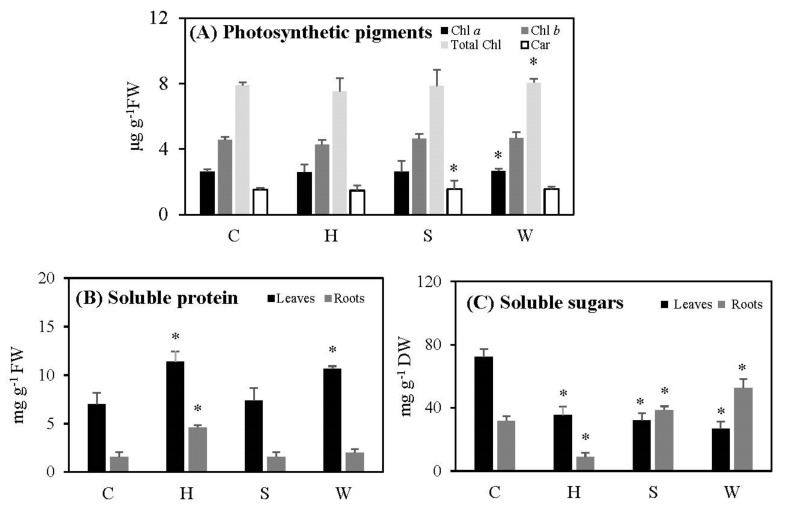
The effect of gradually increased short-term abiotic stresses on the photosynthetic pigments (**A**), total soluble proteins (**B**), and total soluble sugars (**C**) in cowpea plants. Vertical bars represent means ± standard deviations (*n* = 3). Asterisk (*) designates significant changes according to the Student’s *t*-test results between the control and the treatment at *p* < 0.05. Chl: chlorophyll; Car: carotenoids; C: control; H: post-hypoxia; S: salinity; W: water deficit.

The levels of photosynthetic pigments of cowpea leaves—including total chlorophyll, chlorophyll *a*, chlorophyll *b*, and carotenoids—did not show significant differences following hypoxia; the levels of carotenoids in salt-stressed leaves and of chlorophyll *a* and total chlorophyll in water-deficient leaves showed a small but significant increase as compared to the controls (Figure 1A).

Soluble protein levels increased significantly in leaves treated with post-hypoxia and water deficit, by 1.6 and 1.5 times relative to their controls, respectively. Roots followed the same pattern as leaves, but to a lesser extent. However, there were no significant differences in leaves or roots following salt stress compared to controls (Figure 1B).

In comparison to controls, total soluble sugar levels reduced significantly following post-hypoxia in both treated leaves and roots (Figure 1C). While this parameter declined dramatically in leaves after salt stress, it increased in roots under both control conditions and salt stress (Figure 1C). When there was a water shortage, soluble sugar levels in leaves dropped dramatically, while they rose significantly in roots (Figure 1C).

### 2.4. NO Production, Lipid Peroxidation, and Cell Damage Following the Three Treatments

**Figure 2:**
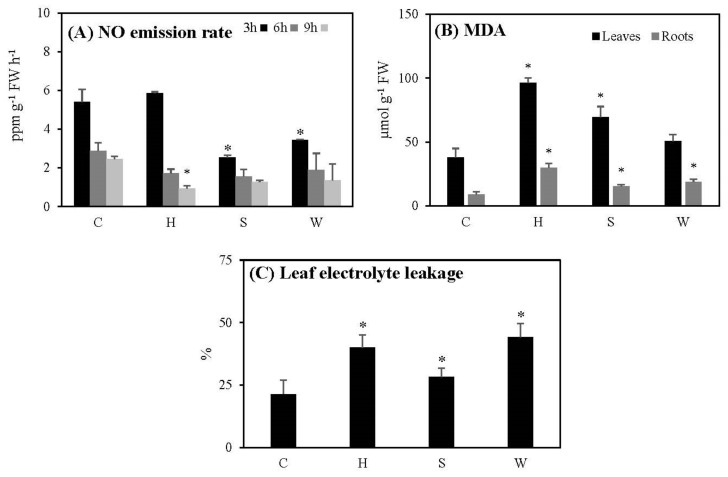
The effect of gradually increased short-term abiotic stresses on the nitric oxide (NO) emission rates (**A**), lipid peroxidation (in terms of MDA) (**B**) and leaf electrolyte leakage (**C**) of the cowpea plants. Vertical bars represent means ± standard deviations (*n* = 3). Asterisk (*) designates significant changes according to the Student’s *t*-test results between the control and the treatment at *p* < 0.05. C: control; H: post-hypoxia; S: salinity; W: water deficit.

The NO emission rates of cowpea plants in both the control and hypoxically treated plants decreased when the duration of nitrogen gas exposure was increased, and at 9 h the difference became significant (Figure 2A). NO emission rates under salt stress and water deficit decreased significantly at 3 h, and there were no significant differences between the treated plants and the controls at 6 or 9 h (Figure 2A).

Lipid peroxidation levels (in terms of MDA) in both hypoxic and salt-stressed leaves and roots, as well as water-deficient roots, increased significantly with respect to the controls (Figure 2B). Furthermore, electrolyte leakage of leaves increased significantly following all types of stresses in this study (Figure 2C).

### 2.5. Antioxidant Capacity of Cowpea Plants Following Hypoxia and Re-Oxygenation

**Figure 3:**
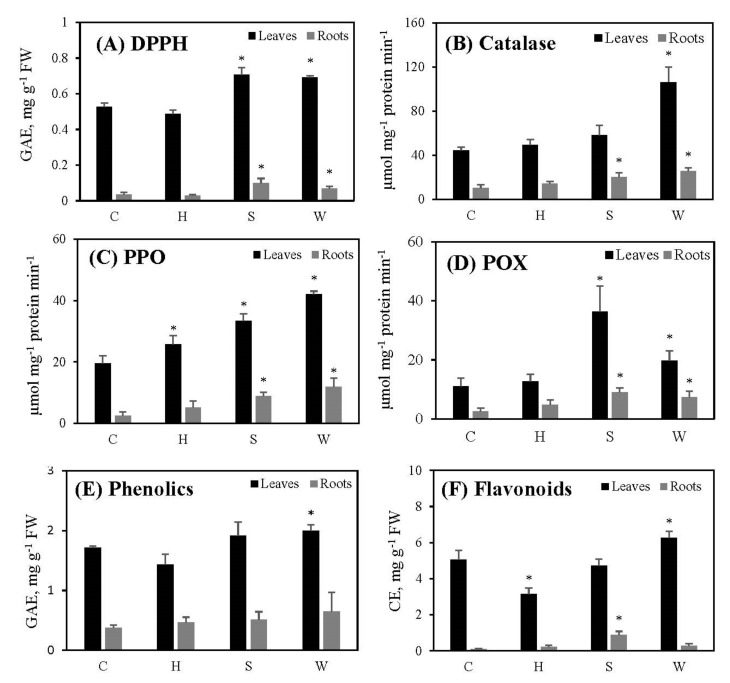
The effect of gradually increased short-term abiotic stresses on the antioxidant capacity of cowpea plants: total antioxidant capacity (DPPH) (**A**), catalase (**B**), polyphenol oxidase (PPO) (**C**), peroxidase (POX) (**D**), phenolics (**E**), flavonoids (**F**). Vertical bars represent means ± standard deviations (*n* = 3). Asterisk (*) designates significant changes according to the Student’s *t*-test results between the control and the treatment at *p* < 0.05. C: control; H: post-hypoxia; S: salinity; W: water deficit.

While the total antioxidant capacity (DPPH) (Figure 3A), catalase (Figure 3B) and peroxidase (POX) (Figure 3D) activities, and phenolic content (Figure 3E) of leaves remained unchanged, polyphenol oxidase (PPO) (Figure 3C) and flavonoid contents (Figure 3F) of leaves decreased significantly following hypoxia. However, all of these parameters in roots remained unchanged following hypoxia (Figure 3A–F).

Total antioxidant capacity (DPPH) (Figure 3A), polyphenol oxidase (PPO) (Figure 3C), and peroxidase (POX) (Figure 3D) activities in leaves increased significantly following salinity; in roots, DPPH activity, all three enzyme activities, and flavonoid levels increased significantly. There were no significant changes in leaf and root phenolics (Figure 3E) in response to salinity.

When the plants were exposed to water deficit treatment, the leaves increased the levels of all antioxidant parameters measured during the study (Figure 3). Furthermore, root DPPH activity and all three enzyme activities increased significantly, while phenolic and flavonoid levels remained unchanged.

## 3. Discussion

Understanding different avenues of plant metabolism and adaptive responses to identify the crops or their traits resistant to abiotic stresses, or to improve their physiological adaptability, is important for global food security. There is limited knowledge of how cowpea plants respond to gradually increasing abiotic stresses when the intensity is changed in a stepwise manner on the same plant; this is because many of the studies are focused on abiotic shocks, where the treatments are carried out separately on different plants, which seems to be an unrealistic phenomenon except in the case of a sudden environmental change [11,12]. Our study reveals how legume plants respond to a stepwise or gradual increase in three abiotic stresses—including post-hypoxia (hypoxia followed by re-oxygenation), salinity, and water deficit, which are common in agriculture—and how these stresses affect the morphology, physiology, and biochemistry of plants.

### 3.1. Soil Parameters

According to our results, it is clear that soil parameters changing significantly following each treatment can pose an effect on the morphological, physiological, and metabolic processes of cowpea plants. Soil oxygen deficiency changes the composition of dissolved gases in the rhizosphere, and affects soil pH and, thus, the nutrient availability and ultimate plant growth [21]. During flooding or waterlogged conditions, soil pH approaches neutrality as the pH of acidic soils increases and the pH of alkaline soils tends to decrease [22]. As seen in our study, two weeks of discontinuously applied hypoxia severely affects soil pH (bringing it to a near-acidic 5.85), as well as the conductivity and total dissolved solids of the soil solution (Table 1). When salinity is increased from 50 to 200 mM, soil electrical conductivity and total dissolved solids increase linearly [23]. In our study, when salinity was gradually increased from 0 to 300 mM NaCl for two weeks, pH, conductivity, and total dissolved solids of the soil solution also increased significantly (Table 1). Similarly, water deficit caused increased soil pH, conductivity, and total dissolved solids (Table 1).

### 3.2. Plant Growth

Some legumes are particularly sensitive to environmental stresses, as they are dependent on nitrogen fixation via symbiotic associations [24]. Flooding decreases transport and translocation of metabolites, rates of photosynthesis, stomatal conductance and rate of transpiration, root and shoot development, and yields [25,26]. Re-exposure to normal oxygen levels following anaerobic pretreatment or natural flooding causes severe injuries in plant tissues and organs due to the generation of superoxide radicals, iron-induced hydroxyl radicals, or other ROS [27]. According to Onwugbuta-Enyi [28], growth parameters of cowpea seedlings—including the relative growth rate of plants, plant height, shoot dry matter, leaf area, and net assimilation rate—did not show significant changes when they were subjected to flooding for 5 weeks. However, our results show that the successive hypoxia treatments during a two-week period resulted in significant decreases in most of the growth and morphological parameters but increased the plant water-use efficiency (Table 2). This indicates that cowpea plants decrease their growth rates due to short-term but continuously increased hypoxia and subsequent reaeration, while they use more water to support the physiological processes of the plant.

Reduction in the rate of leaf surface expansion is the immediate response to increasing salinity, which accounts for ~80% of plant growth reduction, while the remaining 20% of the growth reduction is due to a decrease in stomatal conductance [29]. However, cowpea has been suggested to be used as an alternative crop for salt-affected soils [30]. The application of Na^+^ (in NaCl or in other salts) reduces shoot and root growth in cowpea during the vegetative and reproductive growth of plants [31]. Salinity severely inhibited shoot and root length, leaf relative water content, chlorophylls, the number of branches, pods, and seeds, seed yield, and biomass per plant in lentils [32]. Increased salinity caused harmful effects on the germination rate and seedling growth, including the root length, shoot length, dry matter production, and vigor index of cowpea plants [33]. Abeer et al. [9] reported that salinity reduced growth, biomass, relative water content, and chlorophyll contents in cowpea leaves. In our results, salinity resulted in varying growth responses in cowpea plants, but especially showing reduced growth rates in terms of roots and shoot length, number of leaves, leaf dry and fresh weights, and the fresh weight of whole plant (Table 2). In contrast, plant water-use efficiency decreased while stomatal density increased, showing that cowpea plants were trying to adapt to the increased salinity by decreasing water-use efficiency but maintaining a higher density of stomata.

Although drought, high temperature and longer day-length alone or in combination cause physiological damage and substantial yield reduction in many cowpea cultivars, in general *V. unguiculata* (L.) Walp. was found to be one of the most drought-resistant legumes, grown widely in the semiarid regions [34]. As seen from our results, most of the growth parameters of cowpea plants remained unchanged when watering was gradually reduced for two weeks (Table 1), which suggests that this plant is not severely affected by water deficit in terms of growth. Stomatal control is the major physiological trait to prevent excess water loss in C_3_ plants to avoid water deficit effects causing highly negative water potential [35]. The latter decreases leaf gas exchange and net CO_2_ assimilation rate and thus changes leaf photochemical and biochemical responses and reduces productivity [36,37,38]. According to our results, cowpea plants tend to lose more water by the end of the water deficit period because stomatal density and stomatal index of cowpea leaves increased whereas leaf relative water content and water use efficiency (WUE) decreased (Table 1). Genotypes with high water use index can serve as promising donors for improving water-use efficiency in mungbean grown in soil with poor water holding capacity [38]. The large-scale phenomic studies were found promising for selecting the mungbean genotypes resistant to water stress [39]. In our study, photosynthetic ability remained unchanged which indicates relative resistance of cowpea plants to water stress. Further, it shows that most of the morphological and physiological parameters remain unchanged following all three treatments. 

### 3.3. Photosynthetic Pigments, Sugars, and Protein Contents

Although there was slight increase in chlorophyll *a* and total chlorophyll under water deficit stress, and in carotenoid levels under salt stress, the contents of pigments generally remained largely unchanged after all three treatments, indicating that cowpea plants are relatively resistant to all three stresses when applied gradually over time (Figure 1A). Plant tolerance to stresses, and especially to salt stress, is governed by the accumulation of a large number of low-molecular-weight osmotically active compounds called compatible solutes, or osmolytes, such as sugars, proline, etc. [12]. These osmolytes help to lower the osmotic potential of the cell, enabling the entry of more water, thereby creating a hydration sphere to dilute the accumulated salts [12]. The capacity for osmotic adjustments, which is important for the adaptation to salt stress, is also essential for developing responses to other stresses, e.g., for freezing tolerance [40]. However, under hypoxia, the sugar production was significantly retarded in both leaves and roots, while protein accumulated (Figure 1C). The reduction in soluble sugars in hypoxically treated plants must have occurred due to the lower carbon dioxide levels, as the plants were subjected to nitrogen gas under dark conditions, which eliminates available gases in the soil and atmosphere and limits the availability of light. Furthermore, following re-oxygenation, plants may not have been recovered. When plants were treated with salt stress, leaf sugar accumulation dropped significantly, and protein production remained unaffected, while sugar levels in roots increased (Figure 1B,C). In the leaves of cowpea plants subjected to water deficit, the production of sugars was reduced, while the protein levels accumulated (Figure 1B,C). Thus, sugar production of cowpea leaves was damaged significantly by these continuously increased short-term abiotic stresses, whereas protein production under hypoxia and water deficit remained functional. The reduction in leaf sugar content under all three stresses may also cause a decline in leaf biomass, as discussed above, and as also reported by Banerjee et al. [12]. However, these changes were less pronounced in roots under hypoxia, while salt stress and water loss induced the increases in soluble sugar content in roots.

### 3.4. Nitric Oxide (NO) Production

NO plays an important role as a signaling molecule during plant stress responses [41]. NO also acts as an antioxidant, and helps to neutralize ROS production, reducing oxidative damage [20]. During oxygen deprivation, the mitochondrial electron transport pathway synthesizes more NO by reducing nitrite [42]. In our results, the rates of NO emission in the cowpea plants decreased with time, and showed a significant drop at 9 h (Figure 2A). NO acts also as a signaling molecule, mediating salt stress response in plants [43]. In chickpea and cotton plants, NO regulates the levels of osmolytes and antioxidant enzymes, and delays salt-induced leaf senescence [44]. According to our results, NO production decreased during the increasing salt stress and over time (6 h, 9 h) (Figure 2A). NO directly increases plant tolerance during water deficit, acting as an antioxidant against drought-induced ROS [45]. Nevertheless, our results show that rates of NO emission in cowpea plants under water deficit decreased significantly after 3 h of nitrogen gas exposure, and further decreased with time (Figure 2A). Therefore, under the three stresses of increasing intensity, the ability of cowpea plants to produce NO decreased or did not show a significant change, which may result in the limitations of defense mechanisms related to NO signaling and metabolism.

### 3.5. Cell Damage

A burst of reactive oxygen species occurs immediately after oxygen re-enters the tissues during reaeration, and the degradation of lipids and free fatty acids is facilitated by lipid peroxidation, which is also indicated by increased electrolyte leakage [46]. According to our results, the hypoxia/re-oxygenation and salt stress induced significant leaf and root lipid peroxidation and leaf electrolyte leakage, while lipid peroxidation was more pronounced in roots when the plants were subjected to water deficiency (Figure 2B,C). Nair et al. [47] also recorded similar responses in other cowpea varieties (Pusa Komal and Kanakamony) under drought conditions. According to our results, gradually increased hypoxia, salinity, and water deficit induced more cell damage in both leaves and roots.

### 3.6. Antioxidant Capacity

Plants possess an antioxidant system involving specific enzymes (POX, CAT, SOD, APX, etc.) and non-enzymatic constituents (ascorbate, glutathione, phenolics, etc.) to withstand oxidative damage [48]. Activities of POX, CAT, APX, SOD, and PPO increase in pigeon pea roots under hypoxic conditions created by waterlogging [49]. Reaeration of roots of lupine seedlings after a hypoxic pretreatment caused a higher activity of SOD, CAT, and POX [50]. In our study, except for a significant increase in PPO activity and a reduction in flavonoid levels in leaves, other antioxidant enzymes and compounds of both leaves and roots following hypoxia remained the same as in the controls (Figure 3). This indicates that hypoxically treated cowpea plants could not activate proper antioxidant defense responses; it may also show that plants became better adapted by the end of two weeks of hypoxia/reaeration, reducing their need to engage their antioxidant defense mechanisms. Antioxidant enzymes improve salt tolerance in plants [51], as confirmed by our results showing that total antioxidant capacity (DPPH), PPO and POX in leaves, and DPPH, catalase, PPO, POD, and flavonoid levels in roots were increased following salt stress treatment (Figure 3). Furthermore, low-mass antioxidants (phenolics and flavonoids) do not seem to play a major role in alleviating salt stress in cowpea plants, because their levels in leaves remained unchanged, and showed a significant increase in root flavonoids only. *V. unguiculata* plants develop high antioxidant capacity to survive better under drought-induced oxidative stress, in which PPO, POX, and CAT activities play a major role [47]. According to our results, antioxidant capacity (DPPH) and antioxidant enzyme activities (catalase, PPO, POX) of both leaves and roots increased under water deficit, and low-mass antioxidants such as phenolics and flavonoids played a role in leaves as well (Figure 3). However, phenolics and flavonoids were less pronounced in roots when under water deficit. Therefore, cowpea plants did not improve their antioxidant defense in response to the increased length of hypoxic treatments, or they might have adapted to post-hypoxia, while the defense capacity was maintained properly following the increased salt and water deficit treatments.

## 4. Material and Methods

### 4.1. Plant Materials and Growth Conditions

Healthy seeds of the cowpea (*Vigna unguiculata* (L.) Walp.) cultivar ‘Waruni’ were surface sterilized with 10% NaOCl and washed three times with autoclaved distilled water. Seeds were germinated in Petri dishes containing sterilized filter paper for five days in growth chambers. The fully grown, healthy seedlings of the same sizes were transplanted into 2 L pots filled with equal volumes of a pot mixture (soil: sand: peat = 2:1:1 (*v*/*v*/*v*)). All pots were watered up to field capacity (FC) one day before sowing to allow for draining of excess water; they were arranged in a completely randomized design in growth chambers (Model PGR-15, Conviron, Winnipeg, MB, Canada) with a 14 h photoperiod, temperature of 26 °C/20 °C (light/dark), relative humidity of 50%, and photosynthetic photon flux density of 150 μmol m^−2^ s^−1^. Plants were irrigated regularly, and after two weeks the poorly developed plants were removed from each pot and seedlings (five in one pot) were grown for another week. The initial dry weight of seedlings before the treatments was determined to calculate plant water-use efficiency later. Other plants were subjected to three treatments for two more weeks.

### 4.2. Treatments and Sampling

Pots (containing three-week-old plants) were divided into three experimental groups (hypoxia/re-oxygenation—defined as post-hypoxia, salt stress, and water deficit) and one control group; they were subjected to gradually increasing stress conditions for two weeks. To reach 75% field capacity of pot media, 110 mL of water (alone or dissolved with salt) needed to be added to each individual pot in each treatment. For the water deficit treatment, 110 mL of water was added to each pot except for the water deficit one until the end of the experiment. The amount of watering was reduced every two days for two weeks as the water deficit treatment (110, 100, 80, 50, 10, 5, and 0 mL). For salt stress conditions, plants were treated with different concentrations of sodium chloride (NaCl) solutions, and the concentration was increased every two days for two weeks (10, 30, 60, 100, 180, 200, and 300 mM).

To test the plants under hypoxia and re-oxygenation conditions (post-hypoxia), they were placed in a custom-built, sealed, dark chamber (5 L) under a steady inflow of nitrogen gas at 120 mL min^−1^ (ALPHAGAZ 1 grade, with ~0.001% oxygen). Each pot was watered with the same amount of water (110 mL) regularly, as in the control. Unidirectional air valves faced outwards in the openings to maintain ambient pressure within the chamber while preventing ambient air from entering [52,53]. The chamber was covered to prevent the entrance of any light, in order to stop photosynthetic activities that would give rise to oxygen. Plants were exposed to nitrogen gas on each day, and the exposure time was increased every two days (0, 1, 2, 3, 4. 5, and 6 h). Plants were flushed with regular air and exposed to light conditions during the rest of the day. As the control treatment, plants were directly sampled from the growth chamber at the end of the two weeks; they had been watered regularly with the same amount of water (110 mL) as in other treatments.

For physiological evaluation, the pots in each treatment were separated and measurements were conducted in the morning, always starting at 08:00 each day. To perform the biochemical analysis, the rest of the pots were used, and the fully developed fourth or fifth leaves and the roots were harvested immediately after the treatment, frozen in liquid nitrogen, and stored at −80 °C. The tests were repeated with at least three biological replicates and three technical replicates.

### 4.3. Soil Parameters

Soil pH, electrical conductivity (EC), and total dissolved solids (TDS) in the soil suspensions under each treatment and control were determined at the end of the experiments using a pH meter and a handheld portable EC and TDS meter (E-1 TDS & EC meter, PROSOR), respectively, as described by Bang-Andreasen et al. [54].

### 4.4. Growth Parameters

Fresh weight (FW) and dry weight (DW) of leaves, stems, and roots of 10 plants from each treatment and the control group were determined after measuring the length–width ratio (L/W), and leaf area was roughly estimated by the product of the length and width (L*W = LA) of leaves. Dry weight was determined after oven-drying at 80 °C for 48 h [55]. Plant shoot and root height and number of compound leaves were also recorded.

Plant pigments were extracted into 80% acetone [56]. Chlorophyll *a* (Chl *a*), chlorophyll *b* (Chl *b*), and carotenoid contents were estimated spectrophotometrically at wavelengths of 646.8, 663.2, and 470 nm, respectively. The content of each pigment was calculated as follows:

Chl *a* = (912.25A_663.2_) − (2.79A_646.8_) µg mL^−1^

Chl *b* = (21.5A_646.8_) − (5.1A_663.2_) µg mL^−1^

Carotenoids = [(1000 × (A_470_ − A_720_) − 2.86 × Chl *a*]/221 µg mL^−1^

Total chlorophylls = (8.2* A_663.2_) + (20.2 × A_646.8_) µg mL^−1^

The impression approach was followed to determine leaf stomatal and epidermal cell densities, which were expressed as the number of stomata per unit of leaf area [57]. Twelve fully expanded, mature leaves in each treatment were selected from six different plants (from six pots), and 3–5 counting fields per leaf were randomly selected to determine stomatal and epidermal cell densities [57], using a compound light microscope (Olympus CH-2, CHS) at 400× magnification.

The stomatal density (SD) (number of stomata per leaf area) and stomatal index (SI) were estimated as described by Xu and Zhou [58]:

Stomatal index (SI) = (No of total stomata)/(No of total stomata + No of total epidermal cells) × 100

The water-use efficiency (WUE) and relative water content (RWC) of the individual plant were determined under each stress condition. RWC was determined by using the following standard formula: RWC = (fresh weight − dry weight)/(weight at full turgor − dry weight) × 100. Water content was determined by oven-drying of leaves at 80 °C for 48 h, and full turgor was estimated by keeping the samples overnight in water, as described by Farrant [59]. WUE was calculated as total dry weight divided by total amount of transpiration [60]; the amount of water loss from the pot daily represented the transpiration. Comparable pots, but without plants, were used to correct evaporation.

WUE = (W2 − W1)/∑Evapotranspiration,

where W2 and W1 are the total plant dry weights at the initial and final harvest, respectively.

The total soluble sugar content was determined using the H_2_SO_4_ method [61]. Dry mass of leaves and roots was recorded after drying at 60 °C for 48 h to a constant mass. Leaf powder (0.5 g) was extracted into 10 mL of ethanol-distilled water (8:2 *v*/*v*), and supernatants were collected after centrifuging at 1480× *g* to determine total soluble sugars using H_2_SO_4_. Total soluble protein content was determined as described by Bradford [62].

### 4.5. Nitric Oxide (NO) Emission, Lipid Peroxidation, and Leaf Electrolyte Leakage

Nitric oxide (NO) emission of plants was measured as described by Cochrane et al. [53], by immersing plants in 20 mM HEPES buffer (pH 7.0) and 50 mM sodium nitrate as a nitrogen source. The whole plant was placed in an airtight, dark chamber (to stop photosynthesis, which gives rise to oxygen and reacts with NO) containing two openings on each side, with a steady 120 mL min^−1^ inflow of nitrogen gas through one opening, while the other opening was attached to a chemiluminescent detector (CLD 88 p; ECO PHYSICS AG, Düernten, Switzerland) by a vacuum pump that pulled in air at 120 mL min^−1^ and was connected to an ozone destroyer [7]. A nitric oxide scrubber (ECO PHYSICS AG, Dürnten, Switzerland) was used to absorb any external NO in the measuring gas coming into the system, and gas flow was regulated by flow controllers (Fisher Scientific, Hampton, NH, USA) [63]. The profiles of total NO accumulation by the treated plants after feeding with 50 mM sodium nitrate were averaged every 30 min for five individual plants. As the NO emission starts after ~2 h, maximizes around 3–6 h, and plants standing in the chamber start to deteriorate after ~9–12 h, we calculated the NO emissions after 3 h and up to 9 h.

Lipid peroxidation was measured in terms of malondialdehyde (MDA) content, as described by Heath and Parker [64], using the thiobarbituric acid (TBA) method with minor modifications. Fresh plant biomass (250 mg) was homogenized in 5 mL of 0.1% TCA and centrifuged for 10,000× *g* for 5 min at 4 °C. To a 1 mL aliquot of supernatant, 4 mL of 20% TCA containing 0.5% TBA was added. The mixture was incubated at 95 °C for 30 min, quickly cooled in crushed ice, and centrifuged at 10,000× *g* for 10 min, and then the absorbance was measured spectrophotometrically at 532 nm and 600 nm. The nonspecific absorbance at 600 nm was subtracted from the absorbance at 532 nm, and the concentration of MDA was calculated using an extinction coefficient of 156 mM^−1^ cm^−1^.

Leaf electrolyte leakage was determined as described by Dionisio-Sese and Tobita [65]. Leaf pieces (200 mg, 5 mm diameter) were placed in test tubes containing 10 mL of distilled deionized water; they were incubated in a water bath at 32 °C for 2 h, and the initial electrical conductivity of the medium (EC1) was recorded after cooling down to 25 °C. Then, the tubes were autoclaved at 121 °C for 20 min to release all electrolytes, cooled to 25 °C, and the final electrical conductivity (EC2) was measured. The electrolyte leakage (EL) was calculated by using the formula EL = (EC1/EC2) × 100.

### 4.6. Antioxidant Compounds and Enzyme Activities

Leaf or root samples (0.5 g) were homogenized on ice in 4 mL of ice-cold buffer containing 1 M Tris–HCl (pH 6.8) and 0.3 M sucrose. The homogenates were centrifuged at 13,000× *g* for 30 min at 4 °C. Supernatants were stored at −70 °C and used for enzyme activity assays.

Catalase (CAT; EC 1.11.1.6) activity was measured as described by Guilbault et al. [66]. The reaction buffer consisted of 0.05 M phosphate buffer (pH 7.0) and 3% H_2_O_2_ (*w*/*w*). The reaction was initiated by adding 0.03 mL of plant extract to the reaction buffer solution. The absorbance was measured at 240 nm, and the activity was expressed in units per mg of protein. The unit of activity was defined as 1 μmol of H_2_O_2_ decomposed per min. The molar extinction coefficient for H_2_O_2_ (39.4 M^−1^ cm^−1^) was used to calculate the equivalent catalase activity.

Polyphenol oxidase (PPO; EC 1.10.3.1) activity was determined as described Raymond et al. [67] at 40 °C at 430 nm. The reaction mixture contained 0.2 M sodium phosphate buffer (pH 6.8), 0.2 mL of 0.02 M pyrogallol, and 0.05 mL of the enzyme extract in a total volume of 1 mL.

Peroxidase (POX; EC 1.11.1.7) activity was measured as described by Malik and Singh [68], with a few modifications. The reaction mixture consisted of 3 mL of 0.1 M phosphate buffer (pH 7.0), 0.03 mL of 0.042% H_2_O_2_, 0.05 mL of 20 mM guaiacol, and 0.01 mL of enzyme extract. The increase in absorbance was recorded at 435 nm for a duration of 3 min. POD activity was expressed as units of enzyme activity (U). Units were calculated using a molar absorptivity of 2.66 × 10^4^ M^−1^ cm^−1^ for tetraguaiacol (or 3,3′-dimethoxy-4,4′-biphenoquinone).

DPPH (2,2-diphenyl-1-picrylhydrazyl) radical scavenging activity, total soluble phenolics, and flavonoids were estimated after extracting them into 80 % (*v/v*) acetone with 0.2 % (*m*/*v*) formic acid at a ratio of 1:10, with 8 h of shaking at 4 °C followed by centrifuging at 20,000× *g* for 20 min at 4 °C, as described by Vyas et al. [69]. Total soluble phenolic contents in leaves and roots were determined spectrophotometrically at 725 nm, using Folin–Ciocalteu reagent, as described by Chandrasekara and Shahidi [70], with a few modifications as suggested by Vyas et al. [69]. A gallic acid standard curve was used to determine the total soluble phenolics in terms of gallic acid equivalents (GAE) per root or leaf fresh weight.

Total flavonoid content was measured by the aluminum chloride colorimetric method developed by Zhishen et al. [71], with a few modifications as suggested by Vyas et al. [69]. The absorbance was measured spectrophotometrically at 510 nm, and total flavonoid content was expressed as catechin equivalents (CE) per leaf or root fresh weight.

The DPPH assay was conducted as described by Brand-Williams et al. [72], with a few modifications as suggested by Vyas et al. [69]. The absorbance was recorded spectrophotometrically at 515 nm, and the scavenging capacity was expressed as the percentage of inhibition of DPPH consumption. The gallic acid standard curve was used to express the results as GAE equivalents per tissue fresh weight.

### 4.7. Statistical Analysis

Data are presented as the mean of three independent experiments with the respective SD bars. Statistical significance between means was analyzed with the Student’s *t*-test (*p* < 0.05 was considered significant between controls and treatments, but not between treatments, as stress intensities were not the same), using Minitab version 17 software (Minitab, LLC 17.2.0, State College, PA, USA). The Supplementary Table S1 with *t* and *p* values is provided to assist interpretation of the results.

## 5. Conclusions

Progressive stress application alters soil properties, inducing changes in the morphological, physiological, and metabolic parameters of cowpea plants. Cowpea plants showed varying growth, morphological, and physiological responses under these stresses. Under post-hypoxia, photosynthetic pigments and plant NO production remained initially unchanged, while sugar production and flavonoids in leaves decreased, but leaf and root protein production and cell damage increased. When the salt stress was applied with the increased intensity, sugar production in leaves and NO emission rates decreased, while protein levels remained unchanged. The increased salt application resulted in the induction of cell damage mainly in leaves, despite their higher antioxidant capacity. Water deficit in soil stimulated protein production, induced cell damage, and increased the antioxidant capacity of cowpea plants, while NO production and leaf sugar production declined. All three stresses resulted in stronger responses in leaves than in roots. We conclude that the responses of cowpea plants to the gradually increased abiotic stress factors depend on the type and the degree of stress applied, and on the plant organ. These responses indicate a high plasticity of cowpea plants in the course of their adaptation to environmental changes—a feature that is overlooked in the conventional stress–shock experiments but can be clearly seen when the intensities of the treatments simulate naturally occurring phenomena. Overall, this study provides evidence that plants are able to increase their tolerance to abiotic stress when its intensity changes in a stepwise manner.

## Figures and Tables

**Table 1:** The effects of gradually increased hypoxia, salinity, and water deficit on the parameters of the soil solutions collected following treatments.

Soil Parameter	Control	Post-Hypoxia	Salt Stress	Water Deficit
pH	5.46 ± 0.04	5.85 ± 0.02 *	6.12 ± 0.03 *	5.66 ± 0.03 *
Conductivity (µS/cm)	352.0 ± 5.9	198.8 ± 8.3 *	6332.8 ± 209.3 *	681.8 ± 30.8 *
Total dissolved solids (ppm)	185.8 ± 6.3	100.3 ± 4.7 *	3166.3 ± 104.5 *	382.5 ± 44.7 *

Values represent means ± standard deviations (*n* = 3). Asterisk (*) designates significant changes according to the Student’s *t*-test results between the control and the treatment at *p* < 0.05.

**Table 2:** The effect of gradually increasing short-term abiotic stresses on the morphological and physiological parameters of cowpea plants.

Parameter		Control	Post-Hypoxia	Salinity	Water Deficit
Shoot length (cm)		112.0 ± 6.7	100.9 ± 4.0 *	77.6 ± 8.4 *	109.2 ± 9.6
Root length (cm)		9.2 ± 1.4	6.6 ± 0.8 *	6.7 ± 0.7 *	17.3 ± 4.1 *
Number of compound leaves		10 ± 1.6	11 ± 1.0	7 ± 1.9 *	7 ± 1.3 *
Leaflet area (cm^2^)		97.3 ± 8.4	96.7 ± 8.9	95.4 ± 13.9	97.9 ± 7.5
Fresh weight (g)	Leaves	8.9 ± 1.0	5.7 ± 1.3 *	7.3 ± 0.8 *	5.3 ± 0.7 *
	Roots	1.5 ± 0.4	1.2 ± 0.8	1.5 ± 0.3	1.5 ± 0.3
	Whole plants	18.2 ± 2.1	11.9 ± 2.98 *	15.6 ± 0.9 *	13.2 ± 1.3 *
Dry weight (g)	Leaves	1.1 ± 0.1	0.5 ± 0.1 *	0.9 ± 0.1 *	1.0 ± 0.1
	Roots	0.2 ± 0.1	0.1 ± 0.0	0.2 ± 0.0	0.3 ± 0.1
	Whole plants	2.3 ± 0.3	1.2 ± 0.3 *	2.2 ± 0.1	2.5 ± 0.2
Stomatal density (mm^−2^)		25.0 ± 3.2	25.7 ± 3.6	30.7 ± 6.5 *	53.6 ± 5.9 *
Stomatal index (%)		32.6 ± 4.4	30.5 ± 4.3	35.6 ± 4.1	37.7 ± 4.2 *
Leaf relative water content (%)		89.6 ± 10.1	88.1 ± 4.2	87.9 ± 5.8	47.7 ± 0.4 *
Water-use efficiency (g DW kg^−1^ H_2_O)		2.8 ± 0.1	3.6 ± 0.1 *	2.1 ± 0.1 *	1.7 ± 0.1 *

The values represent means ± standard deviations (*n* = 3). Asterisk (*) designates significant changes according to the Student’s *t*-test results between the control and the treatment at *p* < 0.05.

## Data Availability

All the data are already provided in the main manuscript and in the supplementary table. Contact the corresponding authors if further information or explanation is required.

## Supplementary Materials

The following supporting information can be downloaded at: https://www.mdpi.com/article/10.3390/metabo12010038/s1, Table S1: t and p values of parameters using the Student’s *t*-test: control vs. treatment.
