# Supplementary material for: The Response of Cowpea (Vigna unguiculata) Plants to Three Abiotic Stresses Applied with Increasing Intensity: Hypoxia, Salinity, and Water Deficit
Source: Metabolites. 2022 Jan 4;12(1):38. doi: 10.3390/metabo12010038 (PMC8777733; doi:10.3390/metabo12010038)
Supplement: Supplementary file 1 [file metabolites-12-00038-s001.zip › metabolites-1490097-supplementary.pdf]

Table S1. *t* and *p* values of parameters using the Student's *t*-test: control *vs* treatment.

Parameter		Post-hypoxia		Salinity		Water deficit	
		t value	*p* value	*t* value	*p* value	*t* value	*p* value
Soil parameters	pH	-19.86	0.000	-29.99	0.000	-8.77	0.000
	Conductivity	36.83	0.000	-69.96	0.000	-25.77	0.000
	TDS	26.59	0.000	-69.70	0.000	-10.66	0.000
Morphology	Shoot	3.19	0.019	7.17	0.000	0.53	0.610
	Root	3.59	0.012	3.55	0.012	-4.18	0.014
	Number of compound leaves	-1.20	0.277	2.87	0.024	3.67	0.008
	Leaf area	0.11	0.913	0.26	0.801	-0.13	0.904
Fresh weight	Shoot	4.70	0.001	3.10	0.013	7.30	0.000
	Roots	0.85	0.422	0.43	0.679	0.33	0.751
	Total	4.27	0.003	2.79	0.031	4.93	0.001
Dry weight	Shoot	7.46	0.000	3.07	0.013	1.90	0.089
	Roots	1.18	0.269	-0.04	0.969	-1.23	0.253
	Total	6.77	0.000	0.35	0.739	-1.78	0.113
Physiology	Stomatal density	-0.50	0.622	-2.61	0.021	-14.14	0.000
	Stomatal index	1.22	0.235	-1.69	0.108	-2.80	0.012
	RWC	0.24	0.834	0.25	0.821	7.14	0.019
	WUE	-11.28	0.001	14.12	0.001	21.59	0.000
Pigments	Chl *a*	0.69	0.540	0.50	0.650	-5.74	0.011
	Chl *b*	1.95	0.190	0.88	0.444	-2.41	0.095
	Total Chl	2.13	0.100	0.85	0.459	-3.04	0.038
	Carotenoids	2.68	0.055	-3.09	0.037	-1.47	0.237
Protein	Leaves	-4.80	0.017	-0.36	0.740	-5.67	0.030
	Roots	-8.97	0.003	-0.13	0.904	-1.16	0.329
Sugars	Leaves	8.50	0.005	10.05	0.002	11.24	0.002
	Roots	10.77	0.002	-3.33	0.045	-5.63	0.030
NO	3h	-1.03	0.378	7.11	0.019	4.47	0.021
	6h	2.33	0.146	2.62	0.120	1.99	0.185
	9h	3.11	0.036	2.43	0.136	2.26	0.153
MDA	Leaves	-12.45	0.001	-4.98	0.016	-2.61	0.080
	Roots	-8.70	0.003	-4.14	0.003	-5.20	0.014
Electrolyte	Leaves	-9.74	0.000	-7.12	0.001	-10.15	0.000
DPPH	Leaves	2.06	0.131	-6.74	0.007	-11.44	0.008
	Roots	1.13	0.341	-3.75	0.020	-3.57	0.038
CAT	Leaves	-1.52	0.226	-2.61	0.121	-7.81	0.016
	Roots	-1.90	0.153	-3.61	0.037	-6.59	0.007
POX	Leaves	-0.75	0.507	-4.88	0.039	-3.38	0.043
	Roots	-2.00	0.140	-6.01	0.009	-3.44	0.041
PPO	Leaves	-2.84	0.047	-7.19	0.006	-14.75	0.005
	Roots	-1.83	0.164	-5.84	0.010	-5.20	0.035
Phenolics	Leaves	2.71	0114	-1.54	0.263	-4.61	0.044
	Roots	-1.53	0.224	-1.72	0.227	-1.49	0.274
Flavonoids	Leaves	5.28	0.013	0.86	0.453	-3.40	0.042
	Roots	-2.37	0.141	-7.16	0.019	-2.02	0.181
